# Historical and ongoing inequities shape research visibility in Latin American aquatic mammal paleontology

**DOI:** 10.1038/s42003-025-07863-w

**Published:** 2025-03-21

**Authors:** Ana M. Valenzuela-Toro, Mariana Viglino, Carolina Loch

**Affiliations:** 1 Corporación de Investigación y Avance de la Paleontología e Historia Natural de Atacama (CIAHN Atacama), Caldera, Chile; 2 Instituto Patagónico de Geología y Paleontología (IPGP-CONICET), Puerto Madryn, Argentina; 3 Sir John Walsh Research Institute, Faculty of Dentistry, University of Otago, Dunedin, New Zealand

**Keywords:** Ethics, Careers

## Abstract

Analysis shows that Latin American researchers and women published less on fossil mammals from Latin America than Global North researchers and male counterparts. Papers with more Latin American authors and those written in languages other than English received lower citation rates, highlighting their academic invisibility.

## Teaser

Latin American aquatic mammal fossils offer key insights into the evolution of life in the Southern Hemisphere. However, many fossils are housed in Global North institutions and are studied by overseas researchers. Latin American women researchers also face gender bias that undermines their ability to study fossils, publish, and gain peer recognition. We conducted a bibliographic analysis of studies on fossil aquatic mammals published between 1990 and 2022 to investigate the impacts of scientific colonialism and biases on research and citation patterns in Latin American paleontology. We show that Global North-based researchers published more on Latin American fossils than their Latin American counterparts. Multinational teams authored nearly half of the publications, which are still heavily skewed toward Global North authors. Women led 24% of the articles and were underrepresented in most studies. Papers with more authors received more citations; however, papers with a higher proportion of Latin American authors and published in languages other than English received lower citation rates. The journals’ impact factor affected citation rates for articles authored by Latin American and women, but not by Global North researchers or men. Our paleontology case study shows widespread invisibility for Latin American researchers.

Peer-reviewed publications constitute one of the primary metrics of researchers’ performance, merit, and impact^1–3^. Traditional publication metrics, such as the number of publications and citations, are grounded on the expectation that there are no biases in knowledge production and that scientists have equitable access to research and publication opportunities, with equal opportunities to gain credibility and recognition among peers. However, structural colonialism and gender biases remain common in many scientific fields, affecting the careers of scientists in intricate ways e.g.,^4–6^. Consequently, traditional metrics of publication and recognition may inaccurately assess research performance and may instead reflect existing models of exclusion in the sciences e.g.,^7–9^.

Scientific colonialism is a mindset where the views of the ‘colonizers’ have a higher rank than those of the ‘colonized,’ resulting in the imposition of the colonizers’ perspectives e.g.,^10–15^. Colonial practices are varied, ranging from neglecting the knowledge of local scientists, enforcing English as a “universal language” of science, to defining research priorities. Furthermore, women continue to face challenges in career development ^6,16–19^ despite advances in science representation over the past decade^20^. These gendered experiences intersect with other aspects of researchers’ identities, such as nationality and language, resulting in new dimensions of inequality. As a result, scientists from diverse backgrounds may experience multiple forms of exclusion that disproportionately affect their publication and peer recognition metrics^10^.

Latin America has an exceptional record of fossil aquatic mammals that have contributed to advance our understanding of the evolutionary history of marine vertebrates in the Southern Hemisphere^21^. However, Latin American paleontology has been subject to scientific colonialism, with fossils being illegally collected and exported, leading to permanent deposit and display in overseas institutions^14^. These practices often result in Latin American fossils being studied and published by overseas researchers, with minimal or no involvement of local specialists ^11,14,15^. In addition, for most Latin American scientists, English is a second language, meaning they are likely to spend more time writing and editing articles^22^. Moreover, Latin American women scientists, including paleontologists, routinely experience gender bias, further undermining their ability to research and publish, hampering their chances of becoming leaders in their field^10^.

These practices contribute to the perception that research conducted by Latin American paleontologists, particularly women, has a ‘lower status’ compared to research undertaken by Global North scientists. To test this, we analyzed the publication and citation trends of research in Latin American fossil aquatic mammals from 1990 to 2022. We sought to answer: 1) Who are the researchers publishing on Latin American fossil aquatic mammals and where are they based? 2) What are the citation patterns for publications in the field? and 3) What factors have shaped these citation patterns?

## Results

### Publication trends

**Fig. 1:**
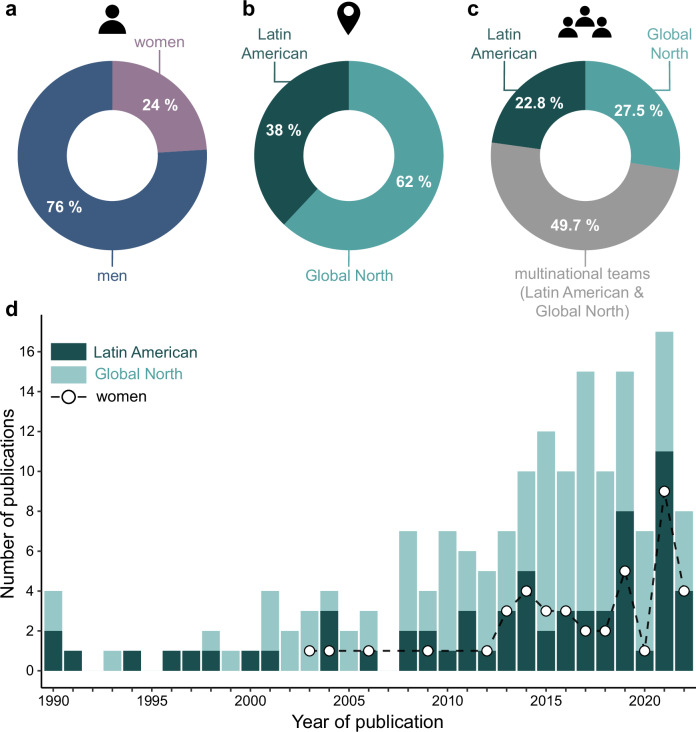
Unbalanced publication trends between 1990 and 2022. Data shows strong regional and gender biases in the publication patterns of research in aquatic mammal fossils from Latin America. **a** Percentage of publications with men as a first author (blue) and women first-author (purple). **b** Percentage of publications with first authors based in Latin America (dark green) or the Global North (turquoise) institutions. **c** Percentage of publications with authors exclusively based in Latin America (dark green), Global North (turquoise), or a combination of both (grey). **d** Number of publications through time with first authors based in Latin America (dark green) or Global North (turquoise) institutions. White dots show the number of publications led by women each year. Note the complete absence of women-led publications before 2003.

A total of 171 publications on fossil aquatic mammals from Latin America were identified, including 156 research articles and 15 reviews (Supplementary Data 1). More than a third of publications (38%) were first authored by researchers based in Latin American institutions (Fig. 1). Almost a third of publications (28.7%) were exclusively authored by Global North-based researchers with no involvement of local scientists. Conversely, nearly half (48.5%) of publications were authored by multinational teams involving at least one Latin American-based researcher. However, more than half (55.4%) of these collaborative publications have a Latin American Index below 0.5 (i.e., number of Latin American authors divided by the total number of authors per paper), showing a bias towards authors from the Global North. Indeed, 39% of these collaborative publications have a Latin American Index equal to or below 0.25, meaning that for every four authors, only one is Latin American. The absolute number of publications with women as first author has increased over time; however, they only represented 24% the articles published between 1990 and 2022 (Fig. 1). Moreover, 86% of the publications had a Women Index less than 0.5 (i.e., number of women authors divided by the total number of authors per paper), showing a strong bias towards publications led by men. Among the 760 authors in our dataset, only 59 (7.8%) are women, with only eight primarily focusing their research on Latin American fossil aquatic mammals. These few scientists are actively researching and publishing, as shown in Supplementary Data 1.

### Citation trends

Publications on fossil aquatic mammals from Latin America received 5184 citations between 1991 and 2022 (Supplementary Data 1 and 2). There were significant differences in the adjusted number of citations received for publications led by researchers based in Latin American vs. Global North institutions (two-way ANOVA: F(1,155) = 20.32, *p* < 0.001), and those led by women vs. men (two-way ANOVA: F(1,155) = 4.52, *p *= 0.035). Publications led by women received only 13.1% of the overall citations. We used generalized linear models to investigate how the number of citations varied over the study period. Two selected models explained 44% and 43% of the variation in the number of citations of overall publications (Table S1). The number of authors positively affected the number of citations (t = 5.78, *p* < 0.001). Conversely, the Latin American Index (t = -2.07; *p* = 0.040) and publishing in a language other than English (t = -3.72; *p* < 0.001) negatively influenced the number of accumulated citations received (Table 1).

**Table 1:** Significant factors influencing the adjusted number of citations per year of articles on fossil aquatic mammals from Latin America for the selected generalized linear models

	Estimate	Standard error	t value	*p* value
**Response variable: citations all publications (*n* = 171)**				
Intercept	0.61	0.14	4.29	< 0.001
Total number of authors	0.043	0.0075	5.78	< 0.001
Latin American Index	−0.31	0.15	−2.07	0.040
Language: Other	−0.42	0.11	−3.72	< 0.001
**Response variable: citations publications first authored by researchers based at Global North (*n* = 106)**				
Intercept	0.62	0.16	3.94	< 0.001
Total number of authors	0.04	0.01	5.19	< 0.001
Latin American Index	−0.43	0.18	−2.37	0.020
Language: Other	−1.23	0.24	−5.17	< 0.001
Journals’ publisher location: Latin America	−0.38	0.17	−2.19	0.031
**Response variable: citations publications first authored by researchers based in Latin America (*n* = 65)**				
Total number of authors	0.06	0.02	3.65	< 0.001
Impact Factor	0.11	0.05	2.05	0.045
Language: Other	−0.30	0.11	−2.65	0.011
**Response variable: citations publications first authored by men (*n* = 130)**				
Intercept	0.67	0.16	4.1	< 0.001
Total number of authors	0.043	0.009	4.73	< 0.001
Latin American Index	−0.4	0.17	−2.39	0.018
Language: Other	−0.52	0.13	−3.86	< 0.001
**Response variable: citations publications first authored by women (*n* = 41)**				
Total number of authors	0.061	0.014	4.34	< 0.001
Impact Factor	0.14	0.035	3.97	< 0.001

Complete estimates (including significant and nonsignificant effects) for all models are shown in Table S6.

Citations of publications first authored by researchers based in Global North institutions were positively affected by the number of authors (t = 5.19, *p* < 0.001), but negatively affected by the Latin American Index (t = -2.37, *p* = 0.020), published in a language other than English (t = -5.17, *p* < 0.001), and publishing in Latin American-based journals (t = -2.19, *p* = 0.0031) (Table 1; Table S2). Similarly, citations of publications first authored by researchers based in Latin America were positively affected by the number of authors (t = 3.65, *p* < 0.001) and the impact factor of the journal (t = 2.05, *p* = 0.045), but negatively impacted by being published in a language other than English (t = -2.65, *p *= 0.011) (Table 1; Table S3). When citation rates of publications led by men and women were analysed separately, we found that different factors influenced them. Citations of publications first authored by men were positively influenced by the total number of authors (t = 4.73, *p *< 0.001) but were negatively influenced by the Latin American Index (t = -2.39, *p *= 0.018) and by publishing in a language other than English (t = -3.86, *p* < 0.001) (Table 1; Table S4). Conversely, the number of authors (t = 4.34, *p* < 0.001) and the impact factor of the journal (t = 3.97, *p* < 0.001) positively affected the citation patterns of articles led by women (Table 1; Table S5).

## Discussion

### Publication disparities

Although the number of publications on fossil aquatic mammals from Latin America has increased over time, pervasive biases are still persistent. Latin American-based researchers first-authored less than 40% of the published studies, whereas nearly a third of publications did not include local researchers. Other findings also deserve closer attention. Nearly half of the publications (48.5%) were conducted by multinational groups led mainly by scientists from Global North institutions. Although the presence of Latin American researchers may indicate inclusiveness and collaborative practices, we showed that this assumption can be misleading. More than half of these articles have an unbalanced authorship favoring Global North researchers. Indeed, we show that in ~40% of these “collaborative” publications, Latin American researchers represent a quarter or less of the number of authors. In most cases, Latin American researchers listed are neither the lead nor the corresponding author of the article, the two most important roles. These practices reflect a casual and unidirectional relationship often established by Global North researchers when conducting research in Latin America e.g.,^12,14,23–26^.

Inadequate funding, lack of research equipment and infrastructure, and unstable scientific landscapes can be decisive factors limiting scientific production and can explain the lower number of publications by Latin American-based researchers^10,27^ (Fig. 1). These factors also lead to the “brain drain” phenomenon commonly seen in Latin American countries e.g.,^28,29^. Indeed, several Latin American researchers studying fossil aquatic mammals have been compelled to work and live in the Global North in recent years (pers. comm.). Many of these scientists thrive abroad (see^21^), suggesting that lower productivity by Latin American researchers is a result of insufficient resources. Nevertheless, additional obstacles stemming from structural biases might worsen research disparities, especially for locally based Latin American paleontologists. The unethical collection of fossils by overseas institutions can exacerbate an already unbalanced scientific production^11,12,14,15^. Requests to examine the type or additional specimens of Latin American fossils are common during peer-review. While scientifically reasonable, these requests often overlook the challenges of accessing local fossils housed in Global North institutions, leading to further publication delays and manuscript rejections. Moreover, researchers whose English is a second language spend more time writing papers in English but are 2.6 times more likely to be rejected based on language^22^. For many Latin American paleontologists, English is a second language, leading to additional language revisions during the peer review process, further delaying publication times. While these issues are not unique to Latin American paleontologists, they contribute to existing resource limitations, further hampering the productivity of scientists in this region.

The number of studies led by women has increased since the first study was published in 2003. Still, papers led by women remain outnumbered by those of men. The underrepresentation of women extends beyond first authorship, as 86% of publications have a Women Index below 0.5, indicating that women account for less than half of the authors listed. This gendered asymmetry in publications likely results from the fewer women researchers studying and publishing on Latin American fossils than men ( < 8% of all authors are women). Nevertheless, while few, these women are actively publishing and are making significant contributions to the field. In fact, half of the authors of the most recent review in Latin American aquatic mammal paleontology^21^ are women, many of whom are based in Latin America. A noteworthy case is observed in Argentina, the country with the largest paleontological community in Latin America. For the past 15 years, women have constituted the majority of active researchers studying fossil aquatic mammals in Argentina. Among the country’s 565 paleontologists, only four—three of whom are women—specialize in fossil aquatic mammals (Argentine Paleontological Association, pers. comm.). Despite being the lead authors of numerous publications in the past decade, their contributions remain significantly underrecognized, as evidenced by low citation rates (Supplementary Data 1).

Other aspects might also have contributed to the low number of publications led by women. Fieldwork is an integral part of paleontological research, allowing for the exploration of fossil sites, the excavation of fossil specimens, and even the expansion or consolidation of collaborative networks. Women often face obstacles related to fieldwork, including challenges ranging from managing periods while in the field, to discrimination, hostile behavior, and, in extreme cases, sexual harassment and assault^30–32^. As a result, women paleontologists, particularly those in Latin America, are less inclined to participate in fieldwork in remote areas. This decreases their opportunities to network and to carry out research and publish findings^33^, perpetuating a cycle of underrepresentation of women in paleontology.

### Drivers for Disparity in Citations patterns

There are marked disparities in citations received by papers on Latin American fossil aquatic mammals. Publications first authored by researchers based in Global North institutions and those led by men received significantly more citations than those by authors based in Latin America and led by women, respectively. Citations not only measure research impact but are also a gauge of scholarly success and reputation e.g.^7,8,34^. Highly cited scholars often receive more invitations to join collaborative projects, have more opportunities to be keynote speakers at conferences and panels, receive more invitations to serve as reviewers for manuscripts and grant applications, thus fueling their career progression e.g.,^33,35,36^. Consequently, the lower citation rates of Latin American paleontologists, particularly women, generates fewer opportunities for collaboration, funding acquisition, research and publishing, further exacerbating challenges and contributing to the notion that Latin American paleontologists will never reach the same academic level as their Global North peers.

The total number of authors positively influenced citation counts for papers on fossil aquatic mammals from Latin America, regardless of the gender and origin of the first’ author (Table 1). Research with several authors usually benefits from a broader range of expertise, leading, in theory, to a richer diversity of ideas and, eventually, higher citation rates e.g.,^37^. While a higher number of authors leads to a higher number of citations, we found that a greater proportion of Latin American-based researchers in the authorship list tends to reduce citations of all papers, including those first authored by men and by researchers from the Global North (Table 1). Likewise, publications in languages other than English had consistently lower citation rates. English is regarded as the “universal language” of science, and as our results show, publications in different languages remain largely overlooked. Publishing in local or Latin American journals also adversely impacted citation rates of articles led by researchers from the Global North. These results are not surprising, as international journals are often considered the prime platforms for publishing ‘rigorous’ research e.g.,^38–40^. Together, these results embody the perception that research produced by Latin American researchers, published in languages other than English, or in local journals has a ‘lower rank’ compared to research undertaken by scientists from the Global North.

Factors driving citation rates of publications on fossil aquatic mammals from Latin America vary across authors. The journal impact factor did not significantly impact citation rates of overall publications or those first authored by Global North researchers or led by men. These results suggest that other factors, such as professional reputation or collegiality, might play a more relevant role in achieving peer recognition through research citation. Conversely, the impact factor significantly influenced citation rates for articles led by researchers based in Latin America and led by women (Table 1). This suggests that to receive peer recognition, papers led by Latin American researchers and women must meet more rigorous publication standards. Moreover, research productivity might reinforce these disparities, because articles authored by more productive researchers tend to receive more citations than those authored by researchers who publish less (i.e., based in Latin Americans and/or led by women)^41^. Due to publication disparities and different standards for recognition, publications by Latin American and women researchers will continue to be overlooked, further limiting their career advancement.

**Fig. 2:**
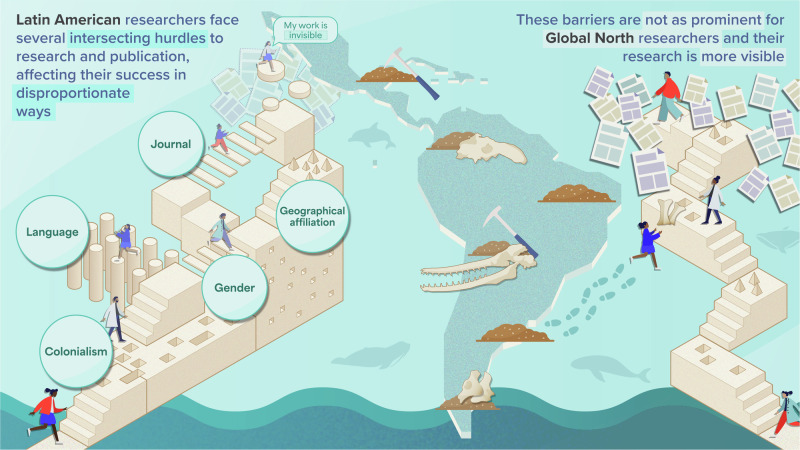
Intersectionality is a pivotal but often overlooked concept that structures research, publication, and citation trends in paleontology. Latin American paleontologists, especially women, encounter several intersecting hurdles to research and publication, which disproportionately affect their visibility, recognition, and career development compared to researchers based in the Global North. Artwork by Melisa Morales.

This paper is a case study of academic invisibility focusing on Latin American paleontology, our field of research and practical expertise. However, the challenges faced by Latin American paleontologists —especially women— unfortunately are not unique. Structural barriers and unconscious bias are pervasive and widespread across various disciplines, affecting the career progression of individuals with non-dominant identities. Researchers with diverse identities live and experience various intersectional challenges tied to their gender, geographic origin, language, and the broader economic and political landscapes of their home countries (Fig. 2). The insights shared here will likely resonate with other regions across the globe and different disciplines, despite regional and discipline-specific particularities.

### Intersectional actions to move forward

We have uncovered how the intersection of science colonialism and gender bias stemming from pervasive cultural structures (including patriarchy) has shaped scientific production and peer recognition over the past three decades. While there is no single solution to these issues, we propose some strategies to move forward.

- Integrity must be a guiding principle for researchers seeking to study overseas fossils. Scientists should commit to strong ethical standards, including conducting lawful excavations, engaging in meaningful collaborations with local researchers, and ensuring fair specimen-based data collection e.g.,^14,42–44^.
- Journals should require authors to provide information on the legal status of reported fossils duringpeer review^14,15^. When possible, editors should invite local experts to review papers reporting or describing Latin American fossils. Latin American paleontologists are likely the most appropriate to give informed feedback on the status of fossils from the region and advise on ethical implications of the study.
- Initiatives promoting accessibility and digitization of fossil collections and associated metadata should be a priority for museum and research institutions, mitigating the harmful effects of Latin American fossils being deposited in overseas collections^45–47^. Although open access to institutional repositories, datasets, and publications has become more common in recent years, data accessibility standards are still uneven among researchers and across disciplines, and are not enforced by journals e.g.,^48–51^. Journals and research institutions should actively enforce open data-sharing practices and more equitable access, ensuring a more permanent availability of these resources to all stakeholders.
- Researchers from Global North institutions conducting paleontology in Latin America should establish symmetrical and respectful relationships with their local counterparts. International collaborations should be synergetic and value diverse perspectives. There must be open and fluid communication about each participant’s expectations, responsibilities, deliverables, and timelines from the earliest stages of the collaboration. There should be transparency in authorship decisions to ensure that contributions from local researchers are accurately reflected in the authorship order. Journals should establish clear authorship policies, including implementing more detailed authorship agreements that specify each author’s contribution and that are agreed upon by each participating author at the time of manuscript submission.
- Journals should provide free language editing services to authors whose English is a second language and encourage publishing by diverse researchers. Still, advances in AI-powered writing and translation tools have benefited science writing for non-native English speakers, and their use should be normalized within an ethical framework. Additionally, editors and reviewers should prioritize scientific content over language and exercise caution when articles on Latin American fossils are exclusively or disproportionally authored by overseas researchers.
- Scientific societies and research institutions should adopt strategies to stimulate women-led research and promote them as role models for young women and early career researchers. The gender gap in science has slightly reduced in some Latin American countries^20^, but women are still underrepresented in senior and decision-making positions e.g.,^52^. Women mentors can challenge gender assumptions in leadership roles, contributing to breaking the “glass ceiling” effect^16^. How can we expect young women to pursue academic careers without visible, relatable, and inspiring models to follow? Journals can also address gender inequities in publications by implementing anonymous peer review, promoting activist choice homophily e.g.,^53^, and citation diversity statements^54^.

## Materials and Methods

### Data collection

We revised the scientific literature describing, reporting, and investigating fossil aquatic mammals from Latin America to elucidate who are the researchers publishing on the topic and where are they based, aiming to unravel the drivers for citation patterns observed. Here we focused on the first author because they often have led the research and whose name is more recognizable among peers^55^. Our search encompassed papers published between 1990 and 2022 (32 years) based on the dataset gathered by ref. ^21^ and^56^ and complemented with further web-based searches using Google Scholar. We focused our search on published peer-reviewed articles and excluded conference abstracts, theses, dissertations, and other sources of information outside traditional publishing channels. We gathered the following information for each article: publication year, first author gender, country and region of affiliation (Latin America or Global North), publication type (original article or review), total number of authors, number of authors from Latin America, number of women among the authors, name, geographic origin, and impact factor of the journal, and language of the article (English or others; including French, Italian, Spanish, and Portuguese). We acknowledge that gender transcends the binary man-woman distinction, can change over time, and should be self-determined. However, due to logistical difficulties in asking each author’s self-perceived gender identity, we used a binary woman-man categorization based on the authors’ first names. For the few gender ambiguous names, we consulted photographs on the authors’ institutional pages. The total number of authors and number of Latin American and women authors were used to compute the ratio of local authors (Latin American Index) and women authors (Women Index) for each article. These indexes ranged from 0 (if there were no Latin American or women researchers involved) to 1 (if all authors were Latin American or women). The Impact Factor of the journals was obtained from Web of Science and complemented with further web-based searches in the journals’ webpages.

We used the search engine Google Scholar to obtain the number of citations of each publication. Only one article was unrecognized by the search engine and thus, excluded from the analyses. Citations were examined for each article and unverifiable or ambiguous citations were excluded. We followed the definition of Global North (Europe, Northern America, Australia, New Zealand, and Eastern and South-eastern Asia) vs. Global South (Central and Southern-Asia, Latin America and the Caribbean, Northern Africa and Western Asia, sub-Saharan Africa, and Oceania) from^57^.

### Statistics and Reproducibility

We used R statistical software version 4.0.3^58^ and the RStudio interface for the analyses. Chi-squared test was used to examine the differences in publication frequency counts of researchers primarily affiliated with institutions from Latin America vs. the Global North and by women vs. men. To account for the significant correlation between the number of citations and time since publication (Spearman, rho = 0.60, *p* < 0.001), we calculated the ratio between the number of citations accumulated by a paper for the number of years since its publication, ranging from 0 to 1. The adjusted number of citations per year was used for subsequent analyses. We also used generalized linear models (GLMs) to examine the drivers of variability in the adjusted number of citations (response variable) using the glm function. The log transformed number of citations per year was used to satisfy the normality assumption. The first author’s gender, geographical region of affiliation (Latin America or Global North), publication category (original article or review), total number of authors, journal publisher location, impact factor, language of the article (English or others; including French, Italian, Spanish, and Portuguese), and Latin American and Women indices were the explanatory variables on the adjusted number of citations per year for each publication. We ran models for the adjusted number of citations using Gaussian distribution. We ranked models based on their Akaike’s information criterion (AIC) using the package AICcmodavg^59^. The models with the lowest AIC values were considered to best fit^60^. Model validation was conducted by plotting residuals versus fitted values.

## Supplementary information

Supplementary Information

Description of Additional Supplementary Materials

Supplementary Data 1

Supplementary Data 2

## Data Availability

All data are available in the main text or the supplementary material.
