# Supplementary material for: Historical and ongoing inequities shape research visibility in Latin American aquatic mammal paleontology
Source: Commun Biol. 2025 Mar 21;8:472. doi: 10.1038/s42003-025-07863-w (PMC11928654; doi:10.1038/s42003-025-07863-w)
Supplement: Supplementary file 1 — Supplementary Information [file 42003_2025_7863_MOESM1_ESM.pdf]

How Historical Inequities Shape Research Visibility in Latin American Aquatic Mammal Paleontology

Ana M. Valenzuela-Toro[1*], Mariana Viglino[2], Carolina Loch[3]

1- Corporación de Investigación y Avance de la Paleontología e Historia Natural de Atacama (CIAHN Atacama), Caldera, Chile.
2- Instituto Patagónico de Geología y Paleontología (IPGP-CONICET), Puerto Madryn, Argentina.
3- Sir John Walsh Research Institute, Faculty of Dentistry, University of Otago, Dunedin, New Zealand.

*Corresponding author: anavalenzuela@ciahn.cl

SUPPLEMENTARY MATERIALS LEGENDS

Supplementary Data 1. List of publications used for the analyses of fossil aquatic mammals from Latin America.

Supplementary Data 2. List of citations for each of the analyzed articles.

Table S1. Generalized linear models examine the first author's gender, geographical region of affiliation (Latin America or Global North), publication category (original article or review), total number of authors, journal publisher location, impact factor of the journal, language of the article (English or others; including French, Italian, Spanish, and Portuguese), and Latin American and Women indices' effect on the adjusted number of citations per year (Gaussian distribution, identity link function) of publications of fossil aquatic mammals from Latin America. The log-transformed number of citations was used to satisfy the normality assumption. K is the number of estimated parameters for each model. AICc is the information criterion for each model. ΔAICc is the difference between the AICc of the corresponding model and the minimum AICc calculated for all models. Model Lik. is the relative likelihood of the model. AICc Wt. indicates the level of support in favor of each model. Cum. Wt. is the cumulative Akaike weights. Exp. Var. is the variability explained by each model.

Table S2. Generalized linear models were used to examine the effect of the gender of the first author, publication category (original article or review), total number of authors, journal publisher location, impact factor, language of the article (English or others; including French, Italian,

Spanish, and Portuguese), and Latin American and Women indices' on the adjusted number of citations per year (Gaussian distribution, identity link function) of publications first authored by researchers based in Global North institutions. The log-transformed number of citations was used to satisfy the normality assumption. K is the number of estimated parameters for each model. AICc is the information criterion for each model. ΔAICc is the difference between the AICc of the corresponding model and the minimum AICc calculated for all models. Model Lik. is the relative likelihood of the model. AICc Wt. indicates the level of support in favor of each model. Cum. Wt. is the cumulative Akaike weights. Exp. Var. is the variability explained by each model.

Table S3. Generalized linear models were used to examine the effect of the gender of the first author, publication category (original article or review), total number of authors, journal publisher location, impact factor, language of the article (English or others; including French, Italian, Spanish, and Portuguese), and Latin American and Women indices' on the adjusted number of citations per year (Gaussian distribution, identity link function) of publications first authored by researchers based in Latin American institutions. The log-transformed number of citations was used to satisfy the normality assumption. K is the number of estimated parameters for each model. AICc is the information criterion for each model. ΔAICc is the difference between the AICc of the corresponding model and the minimum AICc calculated for all models. Model Lik. is the relative likelihood of the model. AICc Wt. indicates the level of support in favor of each model. Cum. Wt. is the cumulative Akaike weights. Exp. Var. is the variability explained by each model.

Table S4. Generalized linear models were used to examine the effect of the geographical region of affiliation (Latin America or Global North), publication category (original article or review), total number of authors, journal publisher location, impact factor, language of the article (English or others; including French, Italian, Spanish, and Portuguese), and Latin American and Women indices' on the adjusted number of citations per year (Gaussian distribution, identity link function) of publications first authored by men of fossil aquatic mammals from Latin America. The log-transformed number of citations was used to satisfy the normality assumption. K is the number of estimated parameters for each model. AICc is the information criterion for each model. ΔAICc is the difference between the AICc of the corresponding model and the minimum AICc calculated for all models. Model Lik. is the relative likelihood of the model. AICc Wt. indicates the level of support in favor of each model. Cum. Wt. is the cumulative Akaike weights. Exp. Var. is the variability explained by each model.

Table S5. Generalized linear models were used to examine the effect of the geographical region of affiliation (Latin America or Global North), publication category (original article or review), total number of authors, journal publisher location, impact factor, language of the article (English or others; including French, Italian, Spanish, and Portuguese), and Latin American and Women indices' on the adjusted number of citations per year (Gaussian distribution, identity link function) of publications first authored by women of fossil aquatic mammals from Latin America. The log-

transformed number of citations was used to satisfy the normality assumption. K is the number of estimated parameters for each model. AICc is the information criterion for each model. ΔAICc is the difference between the AICc of the corresponding model and the minimum AICc calculated for all models. Model Lik. is the relative likelihood of the model. AICc Wt. indicates the level of support in favor of each model. Cum. Wt. is the cumulative Akaike weights. Exp. Var. is the variability explained by each model.

Table S6. Estimates of the adjusted number of citations per year of articles on fossil aquatic mammals from Latin America for the selected generalized linear models. Significant variables are in bold.

Table S1. Generalized linear models were used to examine the effect of the first author's gender, geographical region of affiliation (Latin America or Global North), publication category (original article or review), total number of authors, journal publisher location, impact factor, language of the article (English or others; including French, Italian, Spanish, and Portuguese), and Latin American and Women indices' on the adjusted number of citations per year (Gaussian distribution, identity link function) of publications of fossil aquatic mammals from Latin America. The log-transformed number of citations was used to satisfy the normality assumption. K is the number of estimated parameters for each model. AICc is the information criterion for each model. ΔAICc is the difference between the AICc of the corresponding model and the minimum AICc calculated for all models. Model Lik. is the relative likelihood of the model. AICc Wt. indicates the level of support in favor of each model. Cum. Wt. is the cumulative Akaike weights. Exp. Var. is the variability explained by each model.

**Models' explanatory variables**	**K**	**AICc**	**Δ AICc**	**ModelLik**	**AICcWt**	**LL**	**Cum.Wt**	**Exp. Var. (%)**	**Model fit**
Gender of the 1st author + Geographical region of affiliation of the 1st author + Total number of authors + Latin American Index + Women Index + Impact Factor + Language of the article + Journals' publisher location	10	115.03	0.00	1.00	0.52	-46.77	0.52	43.70	$\chi^2(8) = 13.02$, p = 0.00
First author gender + Geographical region of affiliation of the 1st author + Total number of authors + Latin American Index + Women Index + Impact Factor + Language of the article	9	116.05	1.02	0.60	0.31	-48.42	0.83	42.50	$\chi^2(7) = 12.67$, p = 0.00
First author gender + Geographical region of affiliation of the 1st author + Total number of authors + Latin American Index + Women Index + Impact Factor +	11	117.28	2.25	0.32	0.17	-46.74	1.00	43.70	$\chi^2(9) = 13.02$, p = 0.00

Language of the article + Journals' publisher location + Publication category									
First author gender + Geographical region of affiliation of the 1st author + Total number of authors + Latin American Index + Women Index + Impact Factor	8	140.34	25.31	0.00	0.00	-61.69	1.00	32.10	$\chi^2(6) = 9.56$, $p = 0.00$
First author gender + Geographical region of affiliation of the 1st author + Total number of authors + Latin American Index	6	141.26	26.23	0.00	0.00	-64.36	1.00	29.80	$\chi^2(4) = 8.87$, $p = 0.00$
First author gender + Geographical region of affiliation of the 1st author + Total number of authors + Latin American Index + Women Index	7	142.68	27.65	0.00	0.00	-63.97	1.00	30.10	$\chi^2(5) = 8.97$, $p = 0.00$
Language of the article	3	146.65	31.62	0.00	0.00	-70.25	1.00	24.40	$\chi^2(1) = 7.26$, $p = 0.00$
First author gender + Geographical region of affiliation of the 1st author + Total number of authors	5	147.96	32.93	0.00	0.00	-68.78	1.00	25.80	$\chi^2(3) = 7.67$, $p = 0.00$
Total number of authors	3	158.45	43.42	0.00	0.00	-76.15	1.00	18.60	$\chi^2(1) = 5.52$, $p = 0.00$
Journals' publisher location	3	159.82	44.79	0.00	0.00	-76.83	1.00	17.80	$\chi^2(1) = 5.31$, $p = 0.00$
Latin American Index	3	171.72	56.69	0.00	0.00	-82.78	1.00	11.50	$\chi^2(1) = 3.41$, $p = 0.00$

First author gender + Geographical region of affiliation of the 1st author	4	173.68	58.65	0.00	0.00	-82.71	1.00	11.60	$\chi^2(2) = 3.44, p = 0.00$
Geographical region of affiliation of the 1st author	3	176.09	61.06	0.00	0.00	-84.97	1.00	9.00	$\chi^2(1) = 2.68, p = 0.00$
Impact Factor	3	184.42	69.39	0.00	0.00	-89.13	1.00	4.10	$\chi^2(1) = 1.22, p = 0.01$
1 (null model)	2	188.99	73.96	0.00	0.00	-92.45	1.00	0.00	$\chi^2(0) = -0.00$, p = NA
Publication category	3	189.66	74.63	0.00	0.00	-91.75	1.00	0.90	$\chi^2(1) = 0.26, p = 0.24$
First author gender	3	190.96	75.93	0.00	0.00	-92.40	1.00	0.00	$\chi^2(2) = 3.44, p = 0.00$
Women Index	3	191.04	76.01	0.00	0.00	-92.44	1.00	0.00	$\chi^2(1) = 0.01, p = 0.87$

Table S2. Generalized linear models were used to examine the effect of the gender of the first author, publication category (original article or review), total number of authors, journal publisher location, impact factor, language of the article (English or others; including French, Italian, Spanish, and Portuguese), and Latin American and Women indices' on the adjusted number of citations per year (Gaussian distribution, identity link function) of publications first authored by researchers based in Global North institutions. The log-transformed number of citations was used to satisfy the normality assumption. K is the number of estimated parameters for each model. AICc is the information criterion for each model. ΔAICc is the difference between the AICc of the corresponding model and the minimum AICc calculated for all models. Model Lik. is the relative likelihood of the model. AICc Wt. indicates the level of support in favor of each model. Cum. Wt. is the cumulative Akaike weights. Exp. Var. is the variability explained by each model.

**Models' explanatory variables**	**K**	**AICc**	**Δ AICc**	**ModelLik**	**AICcWt**	**LL**	**Cum.Wt**	**Exp. Var. (%)**	**Model fit**	**Models' explanatory variables**
Gender of the 1st author + Total number of authors + Latin American Index + Women Index + Impact Factor + Language of the article + Journals' publisher location	9	73.10	0.00	1.00	0.46	-26.56	0.46	44.68	$\chi^2(7) = 8.08$, p = 0.00	
Gender of the 1st author + Total number of authors + Latin American Index + Women Index + Impact Factor + Language of the article + Journals' publisher location + Publication category	10	73.25	0.15	0.93	0.42	-25.41	0.88	45.93	$\chi^2(8) = 8.31$, p = 0.00	
Gender of the 1st author + Total number of authors + Latin American Index + Women Index + Impact Factor + Language of the article	8	75.75	2.64	0.27	0.12	-29.09	1.00	41.84	$\chi^2(6) = 7.57$, p = 0.00	
Language of the article	3	95.20	22.10	0.00	0.00	-44.48	1.00	21.11	$\chi^2(1) = 3.82$, p = 0.00	
Gender of the 1st author +	7	97.01	23.91	0.00	0.00	-40.90	1.00	26.51	$\chi^2(5) = 4.79$, p = 0.00	

Total number of authors + Latin American Index + Women Index + Impact Factor									
Gender of the 1st author + Total number of authors + Latin American Index	5	97.39	24.29	0.00	0.00	-43.38	1.00	22.81	$\chi^2(3) = 4.13$, p = 0.00
Gender of the 1st author + Total number of authors + Latin American Index + Women Index	6	97.40	24.30	0.00	0.00	-42.25	1.00	24.51	$\chi^2(4) = 4.43$, p = 0.00
Total number of authors	3	102.79	29.69	0.00	0.00	-48.27	1.00	14.96	$\chi^2(1) = 2.71$, p = 0.00
Gender of the 1st author + Total number of authors	4	104.96	31.86	0.00	0.00	-48.27	1.00	14.96	$\chi^2(2) = 2.71$, p = 0.00
Journals' publisher location	3	114.60	41.50	0.00	0.00	-54.18	1.00	4.41	$\chi^2(1) = 0.80$, p = 0.03
Latin American Index	3	116.06	42.96	0.00	0.00	-54.91	1.00	3.02	$\chi^2(1) = 0.55$, p = 0.08
Impact Factor	3	116.35	43.24	0.00	0.00	-55.05	1.00	2.75	$\chi^2(1) = 0.50$, p = 0.09
1 (null model)	2	117.03	43.93	0.00	0.00	-56.46	1.00	0.00	$\chi^2(0) = -0.00$, p = NA
Publication category	3	117.26	44.16	0.00	0.00	-55.51	1.00	1.86	$\chi^2(1) = 0.34$, p = 0.17
Gender of the 1st author	3	119.11	46.00	0.00	0.00	-56.43	1.00	0.06	$\chi^2(1) = 0.01$, p = 0.82
Women Index	3	119.14	46.04	0.00	0.00	-56.45	1.00	0.02	$\chi^2(1) = 0.00$, p = 0.88

Table S3. Generalized linear models were used to examine the effect of the gender of the first author, publication category (original article or review), total number of authors, journal publisher location, impact factor, language of the article (English or others; including French, Italian, Spanish, and Portuguese), and Latin American and Women indices' on the adjusted number of citations per year (Gaussian distribution, identity link function) of publications first authored by researchers based in Latin American institutions. The log-transformed number of citations was used to satisfy the normality assumption. K is the number of estimated parameters for each model. AICc is the information criterion for each model. ΔAICc is the difference between the AICc of the corresponding model and the minimum AICc calculated for all models. Model Lik. is the relative likelihood of the model. AICc Wt. indicates the level of support in favor of each model. Cum. Wt. is the cumulative Akaike weights. Exp. Var. is the variability explained by each model.

**Models' explanatory variables**	**K**	**AICc**	**Δ AICc**	**ModelLik**	**AICcWt**	**LL**	**Cum.Wt**	**Exp. Var. (%)**	**Model fit**
Gender of the 1st author + Total number of authors + Latin American Index + Women Index + Impact Factor + Language of the article	8	30.86	0	1	0.43	-5.96	0.43	53.76	$\chi^2(6) = 4.85$, p = 0.00
Gender of the 1st author + Total number of authors + Latin American Index + Women Index + Impact Factor + Language of the article + Journals' publisher location + Publication category	10	31.34	0.48	0.79	0.34	-3.33	0.77	57.77	$\chi^2(8) = 5.21$, p = 0.00
Gender of the 1st author + Total number of authors + Latin American Index + Women Index + Impact Factor + Language of the article + Journals' publisher location	9	32.55	1.69	0.43	0.18	-5.40	0.96	54.65	$\chi^2(7) = 4.93$, p = 0.00
Gender of the 1st author + Total number of authors +	7	35.66	4.79	0.091	0.039	-9.71	0.99	47.39	$\chi^2(5) = 4.27$, p = 0.00

Latin American Index + Women Index + Impact Factor									
Impact Factor	3	41.98	11.12	0	0	-17.77	1.00	30.53	$\chi^2(1) = 2.75$, $p = 0.00$
Language of the article	3	42.99	12.12	0	0	-18.27	1.00	29.32	$\chi^2(1) = 2.64$, $p = 0.00$
Gender of the 1st author + Total number of authors	4	43.65	12.78	0	0	-17.45	1.00	31.30	$\chi^2(2) = 2.82$, $p = 0.00$
Total number of authors	3	44.62	13.76	0	0	-19.09	1.00	27.29	$\chi^2(1) = 2.46$, $p = 0.00$
Gender of the 1st author + Total number of authors + Latin American Index	5	45.93	15.07	0	0	-17.39	1.00	31.43	$\chi^2(3) = 2.84$, $p = 0.00$
Journals' publisher location	3	46.61	15.74	0	0	-20.08	1.00	24.76	$\chi^2(1) = 2.23$, $p = 0.00$
Gender of the 1st author + Total number of authors + Latin American Index + Women Index	6	48.36	17.49	0	0	-17.35	1.00	31.51	$\chi^2(4) = 2.84$, $p = 0.00$
Gender of the 1st author	3	56.01	25.14	0	0	-24.78	1.00	11.52	$\chi^2(1) = 1.04$, $p = 0.01$
Women Index	3	59.55	28.69	0	0	-26.55	1.00	5.95	$\chi^2(1) = 0.54$, $p = 0.06$
1 (null model)	2	60.88	30.02	0	0	-28.33	1.00	0	$\chi^2(0) = -0.00$, $p =$ NA
Latin American Index	3	61.82	30.96	0	0	-27.69	1.00	2.20	$\chi^2(1) = 0.20$, $p = 0.26$
Publication category	3	63.01	32.14	0	0	-28.28	1.00	0.18	$\chi^2(1) = 0.02$, $p = 0.75$

Table S4. Generalized linear models were used to examine the effect of the geographical region of affiliation (Latin America or Global North), publication category (original article or review), total number of authors, journal publisher location, impact factor, language of the article (English or others; including French, Italian, Spanish, and Portuguese), and Latin American and Women indices' on the adjusted number of citations per year (Gaussian distribution, identity link function) of publications first authored by men of fossil aquatic mammals from Latin America. The log-transformed number of citations was used to satisfy the normality assumption. K is the number of estimated parameters for each model. AICc is the information criterion for each model. ΔAICc is the difference between the AICc of the corresponding model and the minimum AICc calculated for all models. Model Lik. is the relative likelihood of the model. AICc Wt. indicates the level of support in favor of each model. Cum. Wt. is the cumulative Akaike weights. Exp. Var. is the variability explained by each model.

**Models' explanatory variables**	**K**	**AICc**	**Δ AICc**	**ModelLik**	**AICcWt**	**LL**	**Cum.Wt**	**Exp. Var. (%)**	**Model fit**
Geog. region of affiliation of the 1st author + Total number of authors + Latin American Index + Women Index + Impact Factor + Language + Journals' publisher location	9	104.24	0	1	0.57	-42.34	0.57	45.49	$\chi^2(7) = 12.02$, p = 0.00
Geog. region of affiliation of the 1st author + Total number of authors + Latin American Index + Women Index + Impact Factor + Language	8	105.87	1.63	0.44	0.25	-44.32	0.82	43.73	$\chi^2(6) = 11.56$, p = 0.00
Geog. region of affiliation of the 1st author + Total number of authors + Latin American Index + Women Index + Impact Factor + Language + Journals' publisher location + Publication Category	10	106.59	2.35	0.31	0.18	-42.33	1	45.49	$\chi^2(8) = 12.03$, p = 0.00
Geog. region of affiliation of the 1st author +	5	127.11	22.86	0	0	-58.3	1	29.62	$\chi^2(3) = 7.83$, p = 0.00

Total number of authors + Latin American Index									
Geog. region of affiliation of the 1st author + Total number of authors + Latin American Index + Women Index + Impact Factor	7	128.19	23.95	0	0	-56.62	1	31.49	$\chi^2(5) = 8.32$, p = 0.00
Language	3	128.77	24.53	0	0	-61.29	1	26.18	$\chi^2(1) = 6.92$, p = 0.00
Geog. region of affiliation of the 1st author + Total number of authors + Latin American Index + Women Index	6	129.07	24.82	0	0	-58.18	1	29.76	$\chi^2(4) = 7.87$, p = 0.00
Geog. region of affiliation of the 1st author + Total number of authors	4	133.13	28.88	0	0	-62.4	1	24.86	$\chi^2(2) = 6.57$, p = 0.00
Journals' publisher location	3	137.72	33.48	0	0	-65.76	1	20.71	$\chi^2(1) = 5.47$, p = 0.00
Total number of authors	3	145.37	41.13	0	0	-69.59	1	15.70	$\chi^2(1) = 4.15$, p = 0.00
Latin American Index	3	146.06	41.82	0	0	-69.93	1	15.23	$\chi^2(1) = 4.03$, p = 0.00
Geog. region of affiliation of the 1st author	3	148.62	44.38	0	0	-71.21	1	13.48	$\chi^2(1) = 3.56$, p = 0.00
Impact Factor	3	161.49	57.25	0	0	-77.64	1	4.10	$\chi^2(1) = 1.08$, p = 0.02
1	2	164.62	60.37	0	0	-80.26	1	0.00	$\chi^2(0) = -0.00$, p = NA
Women Index	3	166.01	61.77	0	0	-79.91	1	0.56	$\chi^2(1) = 0.15$, p = 0.41
Publication Category	3	166.05	61.8	0	0	-79.92	1	0.53	$\chi^2(1) = 0.14$, p = 0.42

Table S5. Generalized linear models were used to examine the effect of the geographical region of affiliation (Latin America or Global North), publication category (original article or review), total number of authors, journal publisher location, impact factor, language of the article (English or others; including French, Italian, Spanish, and Portuguese), and Latin American and Women indices' on the adjusted number of citations per year (Gaussian distribution, identity link function) of publications first authored by women of fossil aquatic mammals from Latin America. The log-transformed number of citations was used to satisfy the normality assumption. K is the number of estimated parameters for each model. AICc is the information criterion for each model. ΔAICc is the difference between the AICc of the corresponding model and the minimum AICc calculated for all models. Model Lik. is the relative likelihood of the model. AICc Wt. indicates the level of support in favor of each model. Cum. Wt. is the cumulative Akaike weights. Exp. Var. is the variability explained by each model.

**Models' explanatory variables**	**K**	**AICc**	**Δ AICc**	**ModelLik**	**AICcWt**	**LL**	**Cum.Wt**	**Exp. Var. (%)**	**Model fit**
Geog. region of affiliation of the 1st author + Total number of authors + Latin American Index + Women Index + Impact Factor	7	-2.2	0	1	0.61	10.25	0.61	67.27	$\chi^2(5) = 2.24$, p = 0.00
Geog. region of affiliation of the 1st author + Total number of authors + Latin American Index + Women Index + Impact Factor + Language	8	-0.2	2	0.37	0.23	10.98	0.84	68.68	$\chi^2(6) = 2.29$, p = 0.00
Total number of authors	3	2.14	4.34	0.11	0.07	2.33	0.91	47.91	$\chi^2(1) = 1.60$, p = 0.00
Geog. region of affiliation of the 1st author + Total number of authors + Latin American Index + Women Index + Impact Factor + Language + Journals' publisher location	9	3.04	5.24	0.07	0.04	11.23	0.95	69.13	$\chi^2(7) = 2.30$, p = 0.00
Geog. region of affiliation of the 1st author + Total number of authors	4	4.21	6.41	0.04	0.02	2.59	0.98	48.68	$\chi^2(2) = 1.62$, p = 0.00

Geog. region of affiliation of the 1st author + Total number of authors + Latin American Index + Women Index + Impact Factor + Language + Journals' publisher location + Publication Category	10	6.19	8.39	0.02	0.01	11.69	0.99	69.96	$\chi^2(8) = 2.33$, p = 0.00
Geog. region of affiliation of the 1st author + Total number of authors + Latin American Index	5	6.95	9.15	0.01	0.01	2.6	0.99	48.72	$\chi^2(3) = 1.62$, p = 0.00
Impact Factor	3	7.34	9.54	0.01	0.01	-0.27	1	39.30	$\chi^2(1) = 1.31$, p = 0.00
Geog. region of affiliation of the 1st author + Total number of authors + Latin American Index + Women Index	6	9.81	12.01	0	0	2.65	1	48.88	$\chi^2(4) = 1.63$, p = 0.00
Language	3	15.73	17.93	0	0	-4.47	1	22.30	$\chi^2(1) = 0.74$, p = 0.00
Journals' publisher location	3	20.94	23.14	0	0	-7.07	1	9.43	$\chi^2(1) = 0.31$, p = 0.07
Women Index	3	20.99	23.19	0	0	-7.1	1	9.30	$\chi^2(1) = 0.31$, p = 0.07
Publication Category	3	21.68	23.88	0	0	-7.44	1	7.43	$\chi^2(1) = 0.25$, p = 0.11
1	2	21.9	24.1	0	0	-8.76	1	0.00	$\chi^2(0) = -0.00$, p = NA
Latin American Index	3	22.62	24.82	0	0	-7.91	1	4.85	$\chi^2(1) = 0.16$, p = 0.20
Geog. region of affiliation of the 1st author	3	22.82	25.02	0	0	-8.01	1	4.30	$\chi^2(1) = 0.14$, p = 0.23

Table S6. Estimates of the adjusted number of citations per year of articles on fossil aquatic mammals from Latin America for the selected generalized linear models. Significant variables are in bold.

	**Estimate**	**Standard error**	**t value**	***p* value**
**Response variable: citations all publications (n = 171)**				
**Intercept**	**0.61**	**0.14**	**4.29**	**< 0.001**
Gender of the 1st author: men	-0.074	0.1	-0.72	0.47
Geog. region of affiliation of the 1st author: Global North	-0.14	0.13	-1.10	0.27
**Total number of authors**	**0.043**	**0.0075**	**5.78**	**< 0.001**
**Latin American Index**	**-0.31**	**0.15**	**-2.07**	**0.040**
Women Index	-0.068	0.19	-0.36	0.72
Impact Factor	0.0075	0.04	1.88	0.062
**Language: Other**	**-0.42**	**0.11**	**-3.72**	**< 0.001**
Journals' publisher location: Latin America	-0.17	0.09	-1.77	0.078
**Response variable: citations publications first authored by researchers based at Global North (n = 106)**				
**Intercept**	**0.62**	**0.16**	**3.94**	**< 0.001**
Gender of the 1st author: men	-0.15	0.16	-0.99	0.33
**Total number of authors**	**0.04**	**0.01**	**5.19**	**< 0.001**
**Latin American Index**	**-0.43**	**0.18**	**-2.37**	**0.020**
Women Index	-0.44	0.26	-1.68	0.097
Impact Factor	0.006	0.00	1.40	0.17
**Language: Other**	**-1.23**	**0.24**	**-5.17**	**< 0.001**
Journals' publisher location: Latin America	**-0.38**	**0.17**	**-2.19**	**0.031**
**Response variable: citations publications first authored by researchers based in Latin America (n = 65)**				
Intercept	-0.03	0.22	-0.13	0.90
Gender of the 1st author: men	-0.03	0.12	-0.24	0.81
**Total number of authors**	**0.06**	**0.02**	**3.65**	**< 0.001**
Latin American Index	0.04	0.22	0.18	0.86
Women Index	0.14	0.24	0.61	0.55
**Impact Factor**	**0.11**	**0.05**	**2.05**	**0.045**
**Language: Other**	**-0.30**	**0.11**	**-2.65**	**0.011**
**Response variable: citations publications first authored by men (n = 130)**				
**Intercept**	**0.67**	**0.16**	**4.1**	**< 0.001**
Geog. region of affiliation of the 1st author: Global North	-0.24	0.15	-1.61	0.11
**Total number of authors**	**0.043**	**0.009**	**4.73**	**< 0.001**
**Latin American Index**	**-0.4**	**0.17**	**-2.39**	**0.018**
Women Index	-0.14	0.27	-0.53	0.6

Impact Factor	0.0066	0.0042	1.57	0.12
**Language: Other**	**-0.52**	**0.13**	**-3.86**	**< 0.001**
Journals' publisher location: Latin America	-0.23	0.12	-1.94	0.055
**Response variable: citations publications first authored by women (n = 41)**				
Intercept	-0.097	0.21	-0.46	0.65
Geog. region of affiliation of the 1st author: Global North	0.083	0.18	0.45	0.65
**Total number of authors**	**0.061**	**0.014**	**4.34**	**< 0.001**
Latin American Index	0.066	0.22	0.29	0.77
Women Index	-0.025	0.2	-0.13	0.9
**Impact Factor**	**0.14**	**0.035**	**3.97**	**< 0.001**
