# Supplementary material for: Historical and ongoing inequities shape research visibility in Latin American aquatic mammal paleontology
Source: Commun Biol. 2025 Mar 21;8:472. doi: 10.1038/s42003-025-07863-w (PMC11928654; doi:10.1038/s42003-025-07863-w)
Supplement: Supplementary file 2 — Description of Additional Supplementary Materials [file 42003_2025_7863_MOESM2_ESM.pdf]

Description of Additional Supplementary Files

File name: Supplementary Data 1

Description: List of publications used for the analyses of fossil aquatic mammals from Latin America

File name: Supplementary Data 2

Description: List of citations for each of the analyzed articles.
